# *Lactiplantibacillus plantarum* Probio87 supplementation improves functional constipation and is associated with peripheral gene-expression responses related to inflammation and the gut–brain axis: a randomized, double-blind, placebo-controlled trial

**DOI:** 10.3389/fnut.2026.1876944

**Published:** 2026-07-17

**Authors:** Fan Zheng, Yong Yang, Yu Zhan, Zhe Wen Zhang, Guanting Lu, Daoyuan Xie, Si-Ming Zhang, Uma Mageswary, Yeong-Yeh Lee, Min-Tze Liong

**Affiliations:** 1 Deyang People’s Hospital of Chengdu University of Traditional Chinese Medicine, Deyang, China; 2 School of Medical Sciences, Universiti Sains Malaysia, Kota Bharu, Malaysia; 3 Chengdu Integrated TCM & Western Medicine Hospital, Chengdu, Sichuan, China; 4 Key Laboratory of Molecular Biophysics of the Ministry of Education, Hubei Key Laboratory of Bioinformatics and Molecular Imaging, Center for Artificial Intelligence Biology, Department of Bioinformatics and Systems Biology, College of Life Science and Technology, Huazhong University of Science and Technology, Wuhan, Hubei, China; 5 School of Medicine and Life Sciences, Chengdu University of Traditional Chinese Medicine, Chengdu, China; 6 School of Industrial Technology, Universiti Sains Malaysia, Penang, Malaysia; 7 GI Function and Motility Unit, Hospital Pakar Universiti Sains Malaysia, Kota Bharu, Malaysia

**Keywords:** functional constipation, gut–brain axis, *Lactiplantibacillus plantarum* Probio87, probiotic, randomized controlled trial, whole-blood gene expression

## Abstract

**Background:**

Functional constipation is a common disorder of gut–brain interaction that substantially affects bowel function, psychological well-being, and quality of life. Probiotic supplementation represents a microbiota-targeted nutritional strategy for functional constipation, but associated host-response signals remain incompletely understood.

**Methods:**

This single-center, randomized, double-blind, placebo-controlled trial enrolled 104 adults who met the Rome IV criteria for functional constipation. Participants received either *Lactiplantibacillus plantarum* Probio87 or placebo for 8 weeks, followed by a 4-week post-intervention follow-up. The primary endpoint was weekly complete spontaneous bowel movements. Secondary outcomes included Rome IV symptom scores, PAC-QOL, HAMD-24, safety parameters, and exploratory whole-blood gene-expression profiles related to inflammation, immune regulation, neuroendocrine signaling, and gut motility. The trial was registered in the Chinese Clinical Trial Registry (ChiCTR2300075360).

**Results:**

Probio87 supplementation significantly increased weekly complete spontaneous bowel movements compared with placebo at Week 8 and Week 12. Improvements were also observed in Rome IV symptom scores, constipation-related quality of life, and HAMD-24 domains related to anxiety, sleep, and retardation. Exploratory whole-blood gene-expression analysis showed differential host-response signals after intervention, characterized by increased BDNF expression and reduced expression of IL-1β, CD117, CXCR5, IDO1, DBH, and MLNR in the probiotic group relative to placebo. No intervention-related adverse events or clinically relevant abnormalities in hematological, hepatic, or renal safety parameters were observed.

**Conclusion:**

*L. plantarum* Probio87 supplementation was associated with sustained improvement in bowel function, constipation-related quality of life, and psychological well-being in adults with functional constipation. Exploratory peripheral gene-expression findings suggest that inflammatory, immune, neuroendocrine, and motility-related host-response pathways may be involved, although causal mechanisms require further validation.

**Clinical trial registration:**

https://www.chictr.org.cn/showproj.html?proj=205514, identifier (ChiCTR2300075360).

## 1 Introduction

Functional constipation is a common gastrointestinal disorder, characterized by persistently difficult, infrequent, or seemingly incomplete defecation. It is diagnosed clinically using the Rome IV criteria, which emphasize symptoms such as straining, hard stools, sensation of obstruction, and fewer than three spontaneous bowel movements per week, in the absence of an organic cause (1). The global prevalence-is estimated at 10–20%, substantially impairing quality of life, often leading to associated psychological distress, including anxiety and depression (2).

The pathophysiology of functional constipation is multifactorial, involving dysregulated colonic motility, visceral hypersensitivity, and alterations in the gut-brain axis. Emerging evidence suggests that low-grade mucosal inflammation and immune-mediated activations in the enteric nervous system may impair neuromuscular coordination and delay colonic transit (3, 4). Pro-inflammatory cytokines, such as interleukin-1 beta (IL-1β), could perturb enteric neuronal function and smooth muscle contractility, thereby potentially contributing to delayed colonic transit and constipation symptomatic (5). Concurrently, the gut microbiota dysbiosis – characterized by a reduction in short-chain fatty acid producers and an over presentation of potentially pro-inflammatory taxa – may perpetuate inflammatory states and impair intestinal motility (6, 7).

The brain-gut-microbiota axis represents a bidirectional communication network that integrates intestinal, neural, and psychological functions. Dysregulation of this axis may exacerbate both gastrointestinal and emotional disturbances. Stress and chronic inflammation can alter tryptophan metabolism through the kynurenine pathway, reducing serotonin availability and thereby affecting colonic motility and mood regulation (8, 9). Such neuro-immune alterations are further amplified by microbial dysbiosis, which influences vagal and immune signaling, creating a feedback loop of dysmotility and distress. Recent evidence also implicates altered gastrointestinal hormones such as motilin and somatostatin in impaired contractility and transit (10, 11).

Given the limitations of conventional treatments, such as laxatives and prokinetics, which often provide only symptomatic relief and may cause tolerance or electrolyte imbalances, there is a growing interest in microbiome-modulating approaches. Probiotics, defined as live microorganisms that confer a health benefit on the host when administered in adequate amounts, have emerged as a promising therapeutic strategy. Specific strains of *lactobacillius,* such as *L. casei Shirota and L. rhamnosus GG,* have demonstrated efficacy in improving bowel movement frequency and consistency in some clinical studies (12, 13). Nevertheless, strain-specific effects vary widely, highlighting the need to evaluate the distinct functional and mechanistic properties of *L. plantarum* Probio87 in functional constipation. Their mechanisms extend beyond compositional modulation to include enhancement of gut barrier integrity, suppression of pathogenic bacteria, modulation of host immune responses, and the production of bioactive metabolites that influence enteric nervous system signaling and gut motility. Previous studies have demonstrated that *Lactobacillus* strains possess diverse functional properties that enable modulation of host metabolic pathways and physiology, potentially through the production of bioactive metabolites (14). In addition, probiotic strains have been shown to modulate gut microbiota composition and host physiological responses in clinical and preclinical settings, including improvements in immune function and metabolic regulation, highlighting the importance of strain-specific functional characterization (15, 16).

*Lactiplantibacillus plantarum* is a resilient species commonly found in fermented foods and the human gut, noted for its gastrointestinal tolerance and its immunomodulatory properties (17). Beyond gastrointestinal contexts, its clinical use in other human studies has also demonstrated good tolerability. A recent clinical study in HPV-positive women further confirmed its safety profile and systemic tolerability properties (18), supporting its broader applicability in human health. The strain *L. plantarum* Probio87, isolated from human breast milk, has shown health benefits in previous clinical studies (18, 19). These studies highlighted its gastrointestinal resilience and immune-modulatory capacity, supporting its potential application in functional constipation. However, its efficacy and mechanisms particularly concerning gut immunity, inflammation, and the genes expression linked to motility and the gut-brain axis, remain to be clarified in human trials.

Therefore, we conducted a randomized, double-blind, placebo-controlled trial to evaluate the clinical effects of *Lactiplantibacillus plantarum* Probio87 in adults with functional constipation diagnosed according to the Rome IV criteria. As a dietary and microbiota-targeted intervention, probiotic supplementation provides a practical approach for modulating host–microbe interactions without relying on long-term pharmacological therapy. In functional constipation, nutritional strategies such as fiber intake, hydration, physical activity, and probiotic supplementation may act through complementary mechanisms involving microbial metabolism, immune regulation, and gut–brain communication. The primary objective was to assess its effect on weekly complete spontaneous bowel movements, while secondary outcomes included Rome IV symptom scores, constipation-related quality of life, psychological well-being, and safety. In addition, exploratory whole-blood gene-expression analysis was performed to characterize peripheral host-response signals related to inflammation, immune regulation, neuroendocrine signaling, and gut motility.

## 2 Materials and methods

### 2.1 Probiotic and placebo products

The probiotic *Lactiplantibacillus plantarum* Probio87 was previously characterized and shown to possess key probiotic properties, including resistance to acid and bile, absence of antibiotic resistance in accordance with European Food Safety Authority (EFSA) guidelines, mucin adhesion, utilization of fructooligosaccharide (FOS) and galactooligosaccharide (GOS), and antimicrobial activity against common human pathogens, during the strain characterization and safety evaluation of Probio87 (18). Taxonomic identification of *Lactiplantibacillus plantarum* Probio87 was performed before and after the manufacturing process using 16 s rRNA gene sequencing to ensure strain consistency across production batches, and further validated by whole-genome sequencing, which provided definitive genomic confirmation and comprehensive safety characterization. Each placebo sachet contained 100% non-GMO corn starch, while the probiotic sachet contained 9 log/CFU probiotic blended with 90% non-GMO corn starch. Both products were manufactured by Probionic Corp., Republic of Korea under GMP conditions and without the use of any animal sources ingredients. The products were supplied as light-yellow powders and stored below 25 °C, protected from sunlight according to the manufacturer’s recommendations. Participants consumed one sachet daily of probiotic (9 log CFU/day) or placebo, for 8 weeks.

### 2.2 Study population

Written informed consent was obtained from all participants prior to enrollment. Recruitment was conducted at Deyang People’s Hospital. Eligible participants were adults aged 18–60 years who met the Rome IV diagnostic criteria for functional constipation (1). Individuals were excluded if their constipation was secondary to neurological, metabolic, or obstructive disorders, or medication use. Additional exclusion criteria included-psychiatric comorbidities, immunodeficiency, use of immunosuppressive therapy, severe cardiovascular, cerebrovascular, or renal diseases, intestinal malignancies, or gastrointestinal complications such as perforation, obstruction, or bleeding. Subjects were also excluded if they were currently taking antibiotics or had used antibiotics within the previous 4 weeks, or if they were concurrently enrolled in another clinical trial.

### 2.3 Study protocol

This was a double-blind, randomized, placebo-controlled trial. Randomization was conducted upon considering the inclusion and exclusion criteria. Qualified participants were randomized according to a 1:1 ratio to the two arms of the study according to a computer-generated list, assigned to the probiotic or placebo group with individual codes. The randomization list was concealed from all investigators until study completion. This study was conducted according to the Declaration of Helsinki and was approved by the Medical Research and Ethics Committee of DeYang People’s Hospital (approval no.2023–03-001-K02) and was registered at https://www.chictr.org.cn/ (identifier number ChiCTR2300075360).

The required sample size was calculated based on detecting a between-group difference in weekly complete spontaneous bowel movements (CSBM) using a two-sided test with *α* = 0.05 and 95% power. The sample size calculation was based on the pre-specified primary endpoint, the change in weekly Complete Spontaneous Bowel Movements (CSBM). This approach is a fundamental statistical principle to control Type I error and ensure the trial is adequately powered for a single, clinically meaningful hypothesis. An increase of one CSBM per week represents a well-established Minimally Clinically Important Difference (MCID) and aligns with regulatory standards for functional constipation trials. The numerous secondary endpoints, including microbiome and metabolomic analyses, are vital for exploring mechanisms but were not used for powering the study, as this would necessitate an impractically large sample size and is statistically discouraged. A previous study on chronic constipation demonstrated that probiotic intervention significantly increased the weekly frequency of CSBM by 28% compared to placebo, with mean difference of 0.42 CSBM/week and a standard deviation of 1.2 (20). Based on a two-sided alpha of 0.05, and 80% power, a total of 43 patients were needed per group. To account for an estimated 20% dropout, a total of 108 patients is needed (*n* = 54 per group).

To minimize potential confounding effects of diet and lifestyle, all participants received standardized lifestyle recommendations throughout the study. Each participant was advised to consume a daily breakfast containing~50 g of oats as a practical source of dietary fiber; ≥1,500 mL/day of water to accompany fiber intake; and brisk walking ≥30 min/day as moderate aerobic activity. These recommendations were explained during enrollment and uniformly applied to both probiotic and placebo groups. The placebo product (100% non-GMO corn starch) was consistent with dietary-fiber recommendations for constipation management, aligning with current clinical guidelines (21, 22).

### 2.4 Standardized lifestyle and dietary recommendations

To minimize potential confounding from diet and lifestyle, all participants received standardized lifestyle recommendations throughout the study. These included a daily breakfast containing approximately 50 g of oats as a practical dietary-fiber source, water intake of at least 1,500 mL/day, and at least 30 min/day of brisk walking. These recommendations were uniformly applied to both probiotic and placebo groups. Adherence was monitored using participant diaries and returned sachet counts. However, detailed dietary intake was not quantitatively recorded, which is acknowledged as a limitation.

### 2.5 Safety monitoring

#### 2.5.1 Strain genomic safety: virulence and resistance genes

Genomic DNA of *Lactiplantibacillus plantarum* Probio87 was extracted using the STE method. The integrity of the extracted DNA was verified by agarose gel electrophoresis, and its concentration was quantified using a Qubit fluorometer to ensure suitability for long-read sequencing.

Whole-genome sequencing was performed using PacBio Single Molecule Real-Time (SMRT) technology at Beijing Novogene Bioinformatics Technology Co., Ltd., enabling high-quality genome reconstruction and accurate resolution of repetitive and structurally complex regions. Raw sequencing reads were quality-filtered using SMRT Link v8.0 to remove low-quality sequences and artifacts. The filtered reads were subsequently assembled *de novo* using Canu (23), resulting in a single circular, gapless contig indicative of a complete genome assembly.

Genome annotation was performed using Prokka (24), which identifies open reading frames (ORFs) and assigns functional annotations based on curated databases. The predicted coding sequences were translated into protein sequences for downstream analyses and functional characterization.

Antimicrobial resistance (AMR) genes were identified using the Resistance Gene Identifier (RGI v6.0.5) against the Comprehensive Antibiotic Resistance Database (25), enabling detection of both acquired resistance genes and homologous sequences. To further assess the presence of clinically relevant and horizontally transferable resistance determinants, ResFinder (26) was applied using default parameters.

Virulence-associated genes were predicted by aligning protein sequences against the Virulence Factor Database (27) using DIAMOND BLASTp (v2.1.13; Buchfink et al., 2021), with thresholds set at ≥70% sequence identity and coverage. Complementary analysis was conducted using VirulenceFinder (v2.0.5; Joensen et al., 2014), applying more stringent criteria (sequence identity >90% and coverage >60%) to identify well-characterized virulence determinants and minimize false-positive predictions.

Pathogenic potential was further evaluated using PathogenFinder2 (28), which predicts bacterial pathogenic capacity based on genome-derived protein features and protein language models, providing an additional layer of safety assessment to confirm the absence of pathogenic profiles.

#### 2.5.2 Clinical pathology safety

Fasting blood samples were drawn from an antecubital vein directly into a K₂EDTA tube at week-0 and week-8 by medical personnels. Tubes for serum separation were allowed to clot at room temperature before separated by centrifugation. The resulting serum was aliquoted and stored at −80 °C until analysis. All hematological and biochemical analyses were conducted at the accredited clinical laboratory using standard automated analytical systems of Deyang People’s Hospital. Full blood count was performed on fresh whole blood from K₂EDTA tubes, used to assess the cellular components of blood. Renal and liver function tests were performed on thawed serum aliquots, used to evaluate the performance of kidneys via measuring serum metabolites and electrolytes, and to evaluate hepatic integrity based on serum enzymes and proteins, respectively.

#### 2.5.3 Overall safety assessment

Adverse events (AEs) and product tolerability were monitored throughout the intervention and follow-up periods by trained medical personnel. Participants were instructed to report any discomfort, gastrointestinal disturbance, or unexpected symptoms at each visit. Serious adverse events (SAEs) were defined according to ICH-GCP guidelines and were reviewed by the study physician for causality assessment. The overall safety profile of probiotic supplementation was evaluated based on laboratory findings, reported AEs, and genomic screening results.

### 2.6 Analyses

#### 2.6.1 Complete spontaneous bowel movement (CSBM)

Participants were provided with a standardized bowel diary to record their bowel movement patterns over the study period. Participants were instructed to document the date and time of each bowel movement, differentiate between spontaneous and non-spontaneous bowel movements, and assess stool consistency using the Bristol Stool chart. A CSBM was defined as a bowel movement that occurred without the use of laxatives, enemas, suppositories, or other aids, and was associated with a feeling of complete evacuation. Participants were also asked to note any associated symptoms such as straining or incomplete evacuation. The CSBM score was assessed at baseline (W0), week-8 and week-12.

#### 2.6.2 ROME-IV criteria

The Rome IV criteria were employed to diagnose functional constipation and establish baseline characteristics among participants prior to intervention. The translated Chinese version has shown good reliability and validity when used in clinical settings and, has been adapted culturally to ensure content applicability and accuracy within the context of Chinese medical culture and population (29, 30). The Rome IV questionnaire data were calculated by determining the percentage of defecations affected by at least two of the following symptoms: straining, lumpy or hard stools, sensation of incomplete evacuation, sensation of anorectal obstruction, and manual maneuvers. Functional constipation was diagnosed in participants experiencing these symptoms in at least 25% of defecations, having fewer than three spontaneous bowel movements per week, and not meeting criteria for irritable bowel syndrome. It uses a 5-point Likert scale to evaluate symptoms; 1 = not at all, 2 = slightly, 3 = moderately, 4 = quite a bit, and 5 = extremely or a great deal. Sum-score of all items generates a total score, where higher scores indicate a lower quality of life symptoms (1). The ROME-IV questionnaire was assessed at baseline (W0), week-8 and week-12.

#### 2.6.3 Patient assessment of constipation quality of life (PAC-QOL)

Constipation related quality of life was evaluated using the PAC-QOL questionnaire. This self-reported instrument comprises of 28 questions that cover four subscales: worries and concerns (11 items), physical discomfort (4 items), psychosocial discomfort (8 items), and satisfaction (11 items). Each item is rated on a 5-point Likert scale from 0 (not at all) to 4 (extremely). Subscale scores and a total score are calculated by summing the respective items, with higher scores indicating a poorer, more burdensome quality of life (31).

The PAC-QOL has been translated into multiple languages, including Chinese. The Chinese questionnaire has been validated for use in adults and has shown good reliability and validity when used in clinical settings with Chinese populations (31). The PAC-QOL was administered at baseline (W0), week-8, and week-12.

#### 2.6.4 Hamilton Depression Rating Scale-24 (HAMD-24)

The HAMD-24 is a widely used clinician-administered instrument for assessing depressive symptom severity across multiple domains, including anxiety, cognitive disturbance, sleep, retardation, hopelessness, weight changes, and diurnal variation. The scale has been validated in adult populations, and the Chinese version has demonstrated good reliability and validity in clinical settings (32). In the present study, HAMD-24 was used as a secondary outcome measure to assess psychological well-being rather than to diagnose depressive disorders.

The severity of depressive symptoms was assessed using the HAMD-24 questionnaire. The HAMD-24 is a clinician-administered questionnaire designed to quantify the severity of depression in diagnosed participants. It consists of 24 items assessing core depressive symptoms such as depressed mood, feelings of guilt, suicide ideation, insomnia, agitation, anxiety, and somatic symptoms. The scale employs a 5-point Likert scale of 0–4 (or 0–2 for selected items), where0 indicates absent and higher values indicate greater severity. The total score ranges from 0 to 76, interpreted conventionally as follows: <8 = normal; 8–16 = mild depression; 17–23 = moderate depression; ≥24 = severe depression (32). All clinicians administering the HAMD-24 were trained to ensure inter-rater reliability and minimize assessment bias. Ratings were based on semi-structured interviews, and the total score was calculated as the sum of all 24 items. The HAMD-24 questionnaire was assessed at baseline (W0), week-8 and week-12.

#### 2.6.5 Peripheral gene-expression responses to probiotic supplementation

Gene expression analysis was performed as previously described (33, 34). In brief, whole blood collected in K^2^EDTA tubes was lysed in TRIsure reagent (Bioline, London, United Kingdom), following the manufacturer’s instructions prior to extraction of RNA, followed by isopropanol precipitation and ethanol washing. RNA purity and concentration were determined using a Multiskan™ GO Microplate Spectrophotometer (Thermo Scientific). cDNA was synthesized from 1 μg RNA using the RevertAid RT Kit (Thermo Scientific) with random hexamer primers. Quantitative real-time PCR was performed on an Agilent AriaMx system using SensiFAST SYBR Green mix (Bioline), 50 ng cDNA, and gene-specific primers. The primer sequences and corresponding references for each target gene are provided in (Supplementary Table S1). Gene targets were selected based on their reported roles in inflammatory signaling and gut–brain axis regulation (35–38). Melting curve analysis confirmed amplification specificity. The expression of target genes was normalized to the geometric mean of three reference genes (18S rRNA, beta-actin, and GAPDH), and relative fold changes were calculated using the 2^–ΔΔCt method (39, 40). Several housekeeping genes were used due to varying expression levels and compatibility between housekeeping and target genes, which needed independent normalization and optimization. 18S rRNA was used for highly expressed target genes such as BDNF IL-1β and IDO to avoid Ct saturation and maintain linearity. Beta-actin was used for moderately expressed genes such as CD117 and CXCR5 due to its consistent expression across experimental conditions. Meanwhile, GAPDH was selected for genes DBH and MLNR based on matched expression range and minimal variability in Ct values across samples. All statistical comparisons of gene expression were performed using ΔCt values rather than fold-change values. Fold changes were used for graphical presentation and biological interpretation only. For each target gene, log2 fold changes were calculated as the expression level of the probiotic group relative to the placebo group at the corresponding time point using the 2 − ΔΔCt method. Because gene expression was assessed in whole blood at W0 and W8, these findings were interpreted as exploratory peripheral host-response signals rather than direct evidence of intestinal mucosal gene regulation.

### 2.7 Statistical analyses

Data were analyzed using SPSS version 24.0 (SPSS Inc., Chicago, IL). The primary hypothesis evaluated differences between the two groups of probiotic and placebo. Considering the skewed distribution and non-parametric nature of our data, differences of scale data were compared using the Mann–Whitney U test, while categorical data were compared using the Chi-square test. Spearman’s rank correlation was used in non-parametric correlation analyses with rho (*r*) as the correlation coefficient. Minimal clinically important differences (MCID) were predefined for CSBM (≥1.3/week), PAC-QOL (≥0.5 decrease), and HAMD-24 (≥50% reduction) based on previous clinical evidence, to interpret therapeutic relevance beyond statistical significance (41, 42) All tests were two-sided with *p* < 0.05 as considered statistically significant. Data are presented as mean value ± standard error (SE) unless stated otherwise. For gene-expression analyses, between-group comparisons (probiotic versus placebo) were performed on ΔCt values using non-parametric tests, while relative fold changes were calculated using the 2^–ΔΔCt method and presented as log2 fold change of the probiotic group relative to the placebo group for graphical visualization. Given the exploratory nature of the targeted gene-expression panel, these findings were interpreted as supportive host-response signals and were not considered confirmatory mechanistic endpoints.

## 3 Results

### 3.1 Baseline

**Figure 1:**
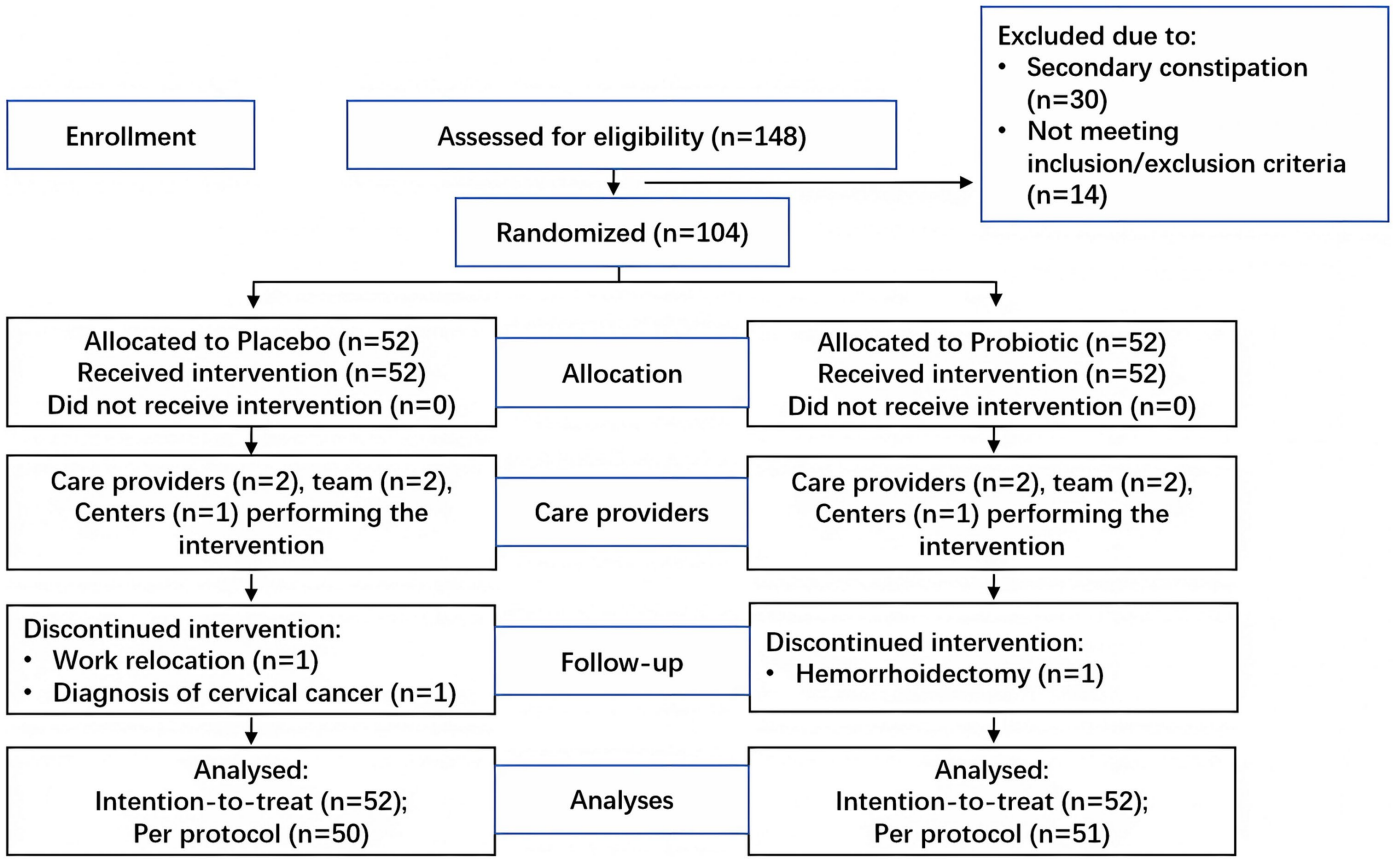
CONSORT flowchart detailing participant recruitment, randomization, allocation, follow-up, and analysis.

A total of 148 participants were assessed for eligibility, of whom 44 were excluded due to secondary constipation or for not meeting the inclusion/exclusion criteria (Figure 1). The remaining 104 participants were randomized to receive either probiotic (*n* = 52) or placebo (*n* = 52); all received their assigned intervention. During the trial, three participants discontinued: one in the probiotic group (due to work relocation) and two in the placebo group (one due to a new diagnosis of cervical cancer and one who underwent a hemorrhoidectomy). All 104 randomized participants were included in the intention-to-treat (ITT) analysis (probiotic: *n* = 52; placebo: *n* = 52). The per-protocol (PP) analysis included 101 participants who completed the study (probiotic: *n* = 51; placebo: *n* = 50). The single-center study was conducted by two designated care providers and their teams. All participants underwent colonoscopy at baseline to exclude organic or secondary causes of constipation. No intervention-related adverse effects or abnormal symptoms were reported, and no participants withdrew due to safety concerns, indicating the treatment was safe and well-tolerated. No participants consumed additional probiotics, antibiotics, or antiviral medications during the study. Adherence was verified through quantitative count of returned unused product sachets at the end of the intervention period, in addition to patient diary logs.

Demographic characteristics were similar between groups at baseline (Table 1). Both groups met the Rome-IV diagnostic threshold of 3.0 and CSBM frequencies below 3.0 per week, confirming the presence of functional constipation with comparable symptom severity.

**Table 1:** Baseline characteristics of constipation patients (*n* = 101) that were randomly assigned to a double-blind administration with either placebo (*n* = 50) or probiotic *Lactiplantibacillus plantarum* Probio87 (*n* = 51) for 8-weeks followed by a 4-week post-intervention observation period.

Baseline characteristics	Placebo (*n* = 50)	Probiotic (*n* = 51)	*p*-value
Sample size (*n*)	50	51	
Gender (%, *n*)			
Male	4 (2)	6 (3)	0.663
Female	96 (48)	94 (48)	
Age (years)	43.78 ± 1.46	43.73 ± 1.56	0.889
BMI (kg/m^2^)	22.23 ± 0.36	21.96 ± 0.35	0.582
Duration of constipation (years)	9.42 ± 0.93	10.19 ± 0.98	0.639
Balloon test (%, *n*)			
Positive	50 (25)	41 (21)	0.601
Negative	50 (24)	49 (25)	
ROME-IV	3.76 ± 0.15	3.49 ± 0.21	0.567
CSBM	2.28 ± 0.13	2.37 ± 0.15	0.399

*p*-value for continuous variables obtained via Mann–Whitney U-test; *p*-value for categorical variables obtained via chi-square test; BMI, body mass index; CSBM, complete spontaneous bowel movement.

### 3.2 Primary outcome-complete spontaneous bowel movements (CSBM)

In this study, the frequency of complete spontaneous bowel movements (CSBM) and its improvement were selected as primary efficacy endpoints to evaluate the therapeutic effect of the probiotic intervention, as increased CSBM frequency is a well-established and reliable marker of treatment efficacy in functional constipation (12, 42). Despite no significant difference at baseline (Placebo: 2.28 ± 0.13, Probiotic: 2.37 ± 0.15; *p* = 0.598), a significant increase in CSBM frequency was observed in the probiotic group compared to the placebo group. This improvement was evident during the 8-week intervention (Placebo: 2.94 ± 0.19, Probiotic: 5.62 ± 0.20; *p* < 0.001) and was sustained at the 12-week post-intervention follow-up (Placebo: 3.08 ± 0.21, Probiotic: 5.06 ± 0.17; *p* < 0.001). Responder analysis, defined as the participants achieving >3 CSBM per week, corroborated these findings. While similar at baseline [Placebo: *n* = 18 (36%), Probiotic: *n* = 24 (47%); *p* = 0.314], significantly more participants in the probiotic group were responders at Week 8 [Placebo: *n* = 32 (64%), Probiotic: *n* = 51 (100%); *p* < 0.001] and Week 12 [Placebo: *n* = 34 (68%), Probiotic: *n* = 51 (100%); *p* < 0.001].

### 3.3 ROME-IV criteria

The severity of constipation symptoms, assessed by the Rome IV questionnaire, is summarized in Table 2. At baseline (W0), no significant differences were observed between the placebo and probiotic groups in any of the ROME IV items or the total score (Table 2). After 8 weeks of intervention, the probiotic group showed significant improvements in strenuous defecation (*p* = 0.010), dry lumpy stool (*p* = 0.010), sensation of incomplete evacuation (*p* = 0.010), sensation of anorectal obstruction (*p* = 0.010), manual maneuvers to facilitate bowel movements (*p* = 0.011), and fewer than three spontaneous bowel movements per week (*p* < 0.001) compared to the placebo group. The total ROME IV score also showed a significant reduction in the probiotic group (*p* < 0.001). During the non-intervention period (W12), the probiotic group maintained these improvements, with significant differences still observed in strenuous defecation (*p* = 0.010), dry lumpy stool (*p* = 0.010), sensation of incomplete evacuation (*p* = 0.010), sensation of anorectal obstruction (*p* = 0.010), manual maneuvers to facilitate bowel movements (*p* = 0.021), and fewer than three spontaneous bowel movements per week (*p* < 0.001). The total ROME IV score remained significantly lower in the probiotic group (*p* < 0.001). The most prevalent changes were sensation of incomplete evacuation, which improved significantly in the probiotic group over 8- and 12-weeks (*p* < 0.05), while the placebo group showed no significant change over time.

**Table 2:** ROME IV items (mean ± SE) for patients with functional constipation and administered with placebo (*n* = 50) or probiotic (*n* = 51).

Item	Placebo					Probiotic					Between-group		
	W0	W8	W12	*p-*value		W0	W8	W12	*p*-value		*p*-value		
				W0–W8	W8-W12				W0–W8	W8-W12	W0	W8	W12
Strenuous	0.86 ± 0.05	0.78 ± 0.06	0.74 ± 0.06	**0.046**	0.157	0.86 ± 0.05	0.08 ± 0.04	0.06 ± 0.03	**<0.001**	0.564	0.968	**0.010**	**0.010**
Dry	0.82 ± 0.06	0.72 ± 0.06	0.70 ± 0.07	0.059	0.705	0.67 ± 0.07	0.14 ± 0.05	0.12 ± 0.05	**<0.001**	0.564	0.080	**0.010**	**0.010**
Sensation of incomplete evacuation	0.64 ± 0.07	0.64 ± 0.07	0.60 ± 0.07	1.000	0.157	0.76 ± 0.06	0.18 ± 0.05	0.06 ± 0.03	**<0.001**	**0.014**	0.172	**0.010**	**0.010**
Sensation of anorectal obstruction	0.70 ± 0.07	0.68 ± 0.07	0.64 ± 0.07	0.564	0.157	0.63 ± 0.07	0.04 ± 0.03	0.00 ± 0.00	**<0.001**	0.157	0.443	**0.010**	**0.010**
Manual maneuvers to facilitate	0.26 ± 0.06	0.12 ± 0.05	0.10 ± 0.04	**0.008**	0.655	0.16 ± 0.05	0.00 ± 0.00	0.00 ± 0.00	**0.005**	1.000	0.204	**0.011**	**0.021**
Fewer than three spontaneous bowel	0.46 ± 0.07	0.34 ± 0.07	0.28 ± 0.06	**0.014**	**0.007**	0.47 ± 0.07	0.00 ± 0.00	0.00 ± 0.00	**<0.001**	**0.000**	0.915	**<0.001**	**<0.001**
ROME IV total score	3.76 ± 0.15	3.28 ± 0.15	3.06 ± 0.17	**0.002**	**0.008**	3.49 ± 0.21	0.43 ± 0.09	0.24 ± 0.07	**<0.001**	**0.019**	0.567	**<0.001**	**<0.001**

W0, baseline; W8, Week 8 (end of intervention); W12, Week 12 (4-week follow-up). Data are presented as mean ± standard error (SE). *p*-values were calculated using the Wilcoxon signed-rank test for within-group comparisons and the Mann–Whitney *U*-test for between-group comparisons. Significant differences (*p* < 0.05) are shown in bold.

### 3.4 Patient assessment of constipation quality of life (PAC-QOL)

**Figure 2:**
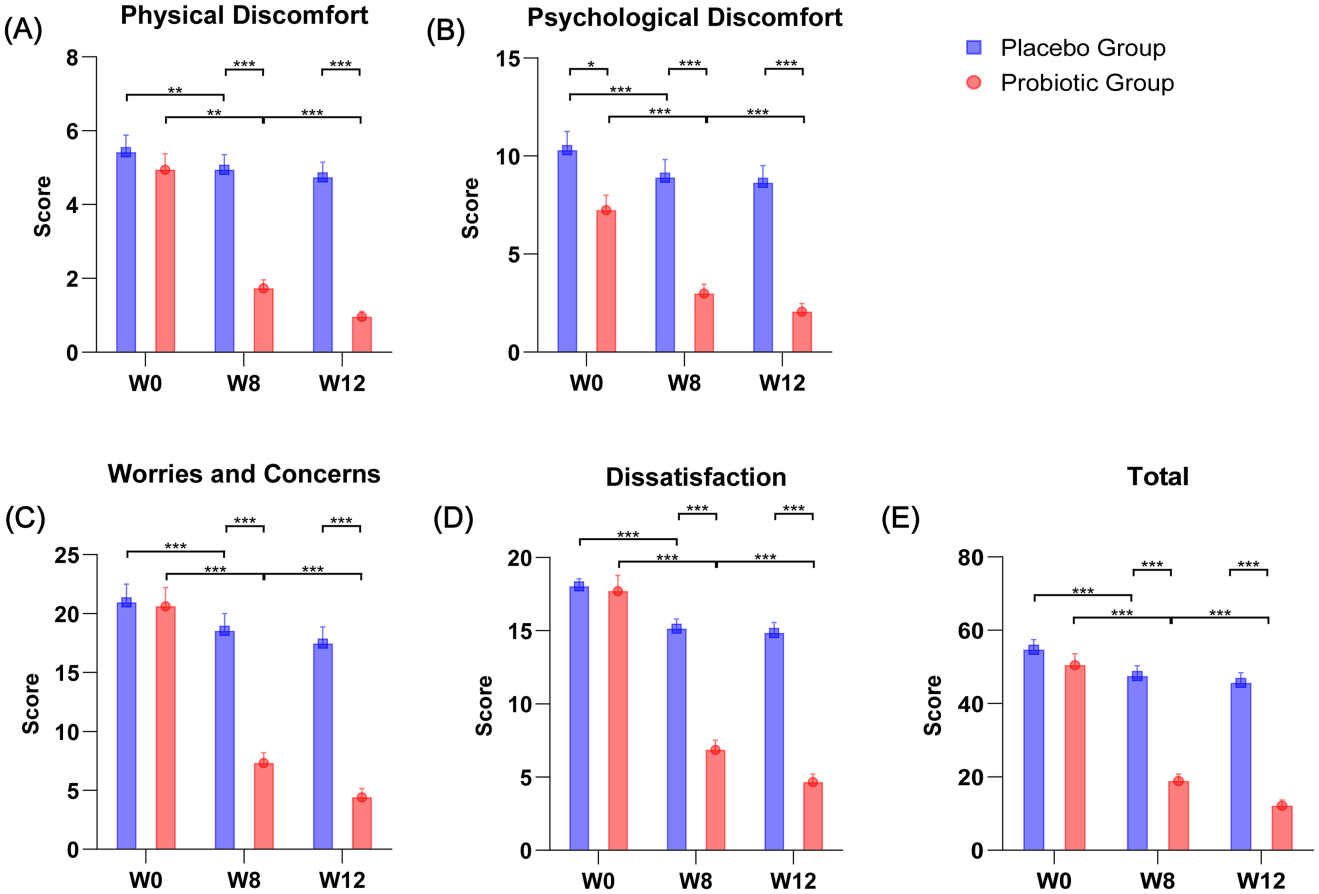
Changes in PAC-QOL domain and total scores in placebo (blue) and probiotic (orange) groups at baseline (W0), Week 8 (W8), and Week 12 (W12). Panels: **(A)** physical discomfort, **(B)** psychological discomfort, **(C)** worries and concerns, **(D)** dissatisfaction, and **(E)** total score. Data are mean ± SE. *p < 0.05, **p < 0.01, ***p < 0.001 vs. placebo at the same time point; #*p* < 0.05 vs. baseline (within-group). Numerical values are presented in Supplementary Table S2.

Quality of life related to constipation was assessed using the validated PAC-QOL questionnaire at baseline (W0), Week 8 (W8), and Week 12 (W12). As shown in Figure 2 (numerical values in Supplementary Table S2) no significant differences were observed between the placebo and probiotic groups for domains of physical discomfort, worries and concerns, dissatisfaction, and the total PAC-QOL score (Supplementary Table S2). After the 8-week intervention (W8), the probiotic group exhibited significant improvements in the domain for physical discomfort, worries and concerns and dissatisfaction (*p* < 0.001) compared to the placebo group. The total PAC-QOL score also showed a significant reduction in the probiotic group (*p* < 0.001). During the post intervention period (W12), these improvements were maintained, with significant differences still observed in these domains (*p* < 0.001). Although baseline score for psychological discomfort was significantly different between groups (*p* = 0.022), the placebo group did not sustain such an effect post intervention while the probiotic group continued to show a significant improvement post intervention (*p* < 0.001).

Overall, probiotic supplementation for 8 weeks, followed by a 4-week observation period, produced significant and sustained improvements in multiple quality-of-life domains among participants with functional constipation.

Alt text: Bar graphs showing PAC-QOL domain and total scores in placebo and probiotic groups at W0, W8, and W12. The figure includes physical discomfort, psychosocial discomfort, worries and concerns, dissatisfaction, and total score. Lower scores indicate better constipation-related quality of life. The probiotic group shows greater reductions in PAC-QOL scores at W8 and W12 compared with placebo, especially in worries and concerns, dissatisfaction, and total score.

### 3.5 Hamilton Depression Rating Scale-24 (HAMD-24)

**Figure 3:**
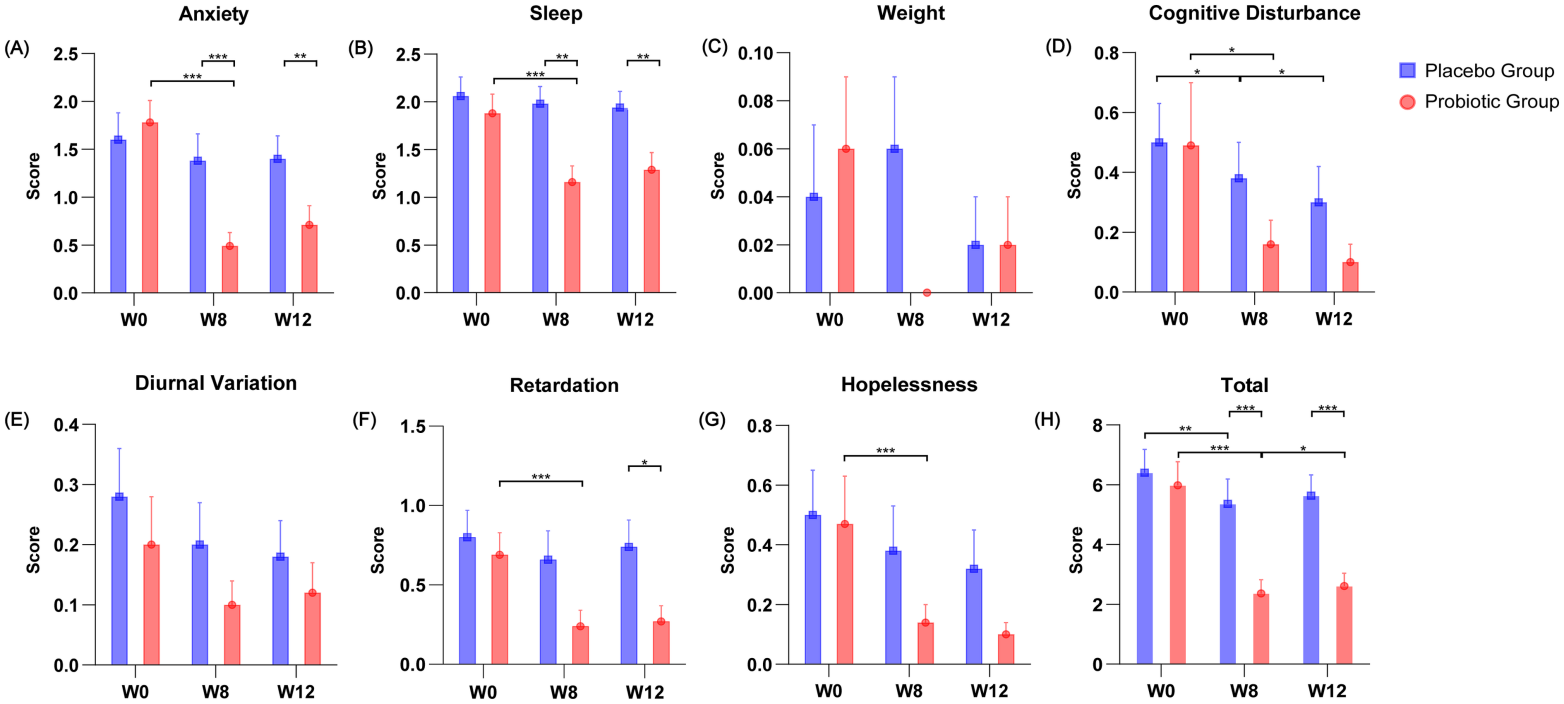
Changes in Hamilton Depression Rating Scale (HAMD-24) subscale scores in placebo and probiotic groups over time. Scores for **(A)** anxiety/somatization, **(B)** sleep, **(C)** weight, **(D)** cognitive disturbance, **(E)** diurnal variation, **(F)** Retardation, **(G)** hopelessness, and **(H)** total score were assessed at baseline (W0), Week 8 (W8), and Week 12 (W12). Data are expressed as mean ± SE.*p < 0.05, **p < 0.01, ***p < 0.001 vs placebo at the same time point.

Psychological well-being was assessed using the HAMD-24 questionnaire at baseline (W0), Week 8 (W8), and Week 12 (W12) (Figure 3). Compared with placebo, participants receiving Lactiplantibacillus plantarum Probio87 showed significant improvements in anxiety/somatization, sleep, retardation, and total HAMD-24 scores at W8 and W12 (numerical values in Supplementary Table S3). Improvements in cognitive disturbance were also observed at selected time points, whereas weight and diurnal variation scores remained largely unchanged between groups. The probiotic group maintained lower total HAMD-24 scores throughout the post-intervention follow-up, indicating sustained benefits on psychological well-being beyond the active supplementation period.

### 3.6 Gene-expression responses to probiotic supplementation

**Figure 4:**
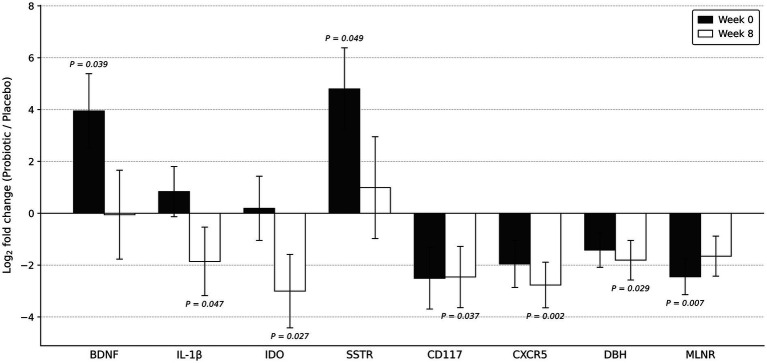
Relative expression of selected host-response genes in the probiotic group using the placebo group as the calibrator (control) at Week 0 and Week 8. Gene expression was quantified by qRT-PCR and expressed as log₂ fold change calculated using the 2−ΔΔCt method, with the placebo group serving as the reference for each time point. Positive values indicate higher expression in the probiotic group, whereas negative values indicate lower expression relative to placebo. Each target gene was normalized to a validated reference gene: 18S rRNA for BDNF, IL-1β, IDO1 and SSTR; β-actin for CD117 and CXCR5; and GAPDH for DBH and MLNR. Data are expressed as mean ± SEM. Statistical comparisons between groups were performed using the Mann–Whitney U test on ΔCt values at the indicated time point. *p*-values are shown for statistically significant comparisons. BDNF, brain-derived neurotrophic factor; IL-1β, interleukin-1 beta; CD117, KIT receptor tyrosine kinase; CXCR5, C-X-C chemokine receptor type 5; IDO1, indoleamine 2,3-dioxygenase 1; DBH, dopamine β-hydroxylase; MLNR, motilin receptor, SSTR (Somatostatin Receptor).

Whole-blood gene-expression analysis revealed differential peripheral host-response signals between the probiotic and placebo groups (Figure 4). At baseline, BDNF and SSTR expression was higher in the probiotic group; however, neither difference was no longer evident after 8 weeks. Following intervention, the probiotic group exhibited lower expression of IL-1β, IDO1, CD117, CXCR5 and DBH relative to placebo.

These transcriptional differences suggest modulation of pathways related to inflammation, immune regulation, neuroendocrine signaling, and gut motility. Specifically, reduced IL-1β and IDO1 expression may reflect attenuation of inflammatory and tryptophan–kynurenine pathway activity, while lower CD117 and CXCR5 expression may indicate altered immune-cell activation and trafficking. Reduced DBH expression further suggests modulation of neuroendocrine and motility-related responses. The baseline differences observed for BDNF, SSTR, and MLNR—which were not sustained at Week 8—may reflect pre-existing group characteristics rather than direct intervention effects, and should be interpreted with caution.

Because gene expression was measured in whole blood rather than intestinal tissue, these findings should be interpreted as exploratory peripheral host-response signals rather than direct evidence of intestinal mechanisms.

### 3.7 Safety monitoring

#### 3.7.1 Genomic safety: antimicrobial resistance and virulence gene profiling

A comprehensive genomic evaluation was conducted to assess antimicrobial resistance and virulence-associated genes in *Lactiplantibacillus plantarum* Probio87. Whole-genome sequencing confirmed a complete, gapless circular genome, and functional annotation revealed a genomic architecture typical of non-pathogenic Lactiplantibacillus strains.

Screening against the CARD database identified two homologous sequences (Supplementary Table S4). One corresponded to D-Ala-D-Ala carboxypeptidase VanY, One corresponded to D-Ala-D-Ala carboxypeptidase VanY, a component of glycopeptide resistance operons in *Enterococcus* species (43). Despite high coverage (95.9%), sequence identity was low (31.93%), suggesting that the detected homolog is more likely a conserved enzyme involved in cell wall metabolism than a functional resistance determinant. The second homolog corresponded to QacJ, a quaternary ammonium compound resistance efflux pump of the small multidrug resistance (SMR) family. Although coverage was high (95.33%), sequence identity was only 40.20%, indicating a distantly related homolog. SMR-family proteins are commonly involved in stress adaptation and membrane homeostasis in non-pathogenic bacteria rather than clinically relevant antimicrobial resistance.

Importantly, ResFinder analysis did not identify any acquired antimicrobial resistance genes (Supplementary Table S4). Because ResFinder specifically identifies clinically relevant acquired resistance genes with potential for horizontal transfer, the absence of such genes further suggests that the detected CARD homologs are unlikely to represent functional or transferable resistance determinants (26).

Virulence factor analysis using VFDB identified homologs of elongation factor Tu (tufA), UTP-glucose-1-phosphate uridylyltransferase (hasC/GalU), and ATP-dependent Clp protease subunit (clpP) (Supplementary Table S4). These genes are highly conserved housekeeping genes that perform essential physiological functions in bacterial growth, metabolism, and stress adaptation. Although included in virulence-related databases because homologs have been reported in certain pathogenic species, they are widely distributed among non-pathogenic bacteria and do not independently indicate pathogenicity.

Consistent with these findings, VirulenceFinder detected no known virulence genes under stringent criteria (Supplementary Table S4). Furthermore, PathogenFinder2 classified the strain as non-pathogenic to humans, with a low mean pathogenicity probability score of 0.063 (Supplementary Table S5). Collectively, these results indicate that *L. plantarum* Probio87 does not harbor clinically relevant antimicrobial resistance or virulence determinants and is unlikely to pose a pathogenic risk.

#### 3.7.2 Clinical pathology safety

To evaluate systemic safety, clinical biochemical parameters were assessed at baseline (W0) and Week 8 (W8). Liver function markers, including alanine aminotransferase (ALT), aspartate aminotransferase (AST), *γ*-glutamyl transferase (GGT), alkaline phosphatase (ALP), total bilirubin, albumin, and total protein, showed no significant differences between the probiotic and placebo groups at either time point (Supplementary Table S6). Similarly, renal function indicators, including serum creatinine, blood urea nitrogen (BUN), and estimated glomerular filtration rate (eGFR), remained stable throughout the study, with all values within established clinical reference ranges. No serious or product-related adverse events were reported during the intervention or follow-up period. These findings indicate that *Lactiplantibacillus plantarum* Probio87 was well tolerated and did not adversely affect hepatic or renal function, consistent with previous clinical evidence supporting the safety of probiotic supplementation in human populations.

## 4 Discussion

Supplementation with *L. plantarum* Probio87 led to a clinically meaningful and sustained improvement in bowel movement frequency compared with placebo, consistent with prior evidence supporting probiotic efficacy in functional constipation.

The persistence of benefit during the post-intervention follow-up suggests that this probiotic may exert longer-term physiological effects through enhanced microbial stability, short-chain fatty acid production, and modulation of intestinal motility pathways (30, 31). These results highlight the potential of *L. plantarum* Probio87 as a safe and effective adjunctive therapy targeting both bowel function and gut ecosystem restoration.

Patients receiving *Lactiplantibacillus plantarum* Probio87 showed a broad improvement in Rome IV symptom domains, reflecting both enhanced bowel function and reduced evacuation difficulty compared with placebo. These findings are consistent with previous clinical studies reporting that probiotic supplementation, particularly with *L. plantarum* strains, can normalize stool consistency, reduce straining, and improve overall colonic motility in patients with chronic constipation (6, 12).

Mechanistically, this improvement is likely linked to modulation of the gut microbiota and attenuation of low-grade intestinal inflammation, which are recognized contributors to functional constipation (7, 44). The observed reduction in Rome IV scores may be attributed to increased short-chain fatty acid (SCFA) production and enhanced mucosal immune regulation, both of which promote propulsive motor activity and restore enteric neuromuscular function (6). The persistent symptom relief during the post-intervention follow-up suggests that *L. plantarum* Probio87 may also enhance microbial stability and gut–brain–motility communication, thereby extending therapeutic effects beyond the treatment period.

Clinically, these outcomes indicate that targeted probiotic supplementation may complement conventional management strategies by improving multiple symptom dimensions simultaneously, including stool form, evacuation ease, and defecation frequency, without adverse events. This supports the potential of *L. plantarum* Probio87 as a safe and sustainable adjunctive approach in functional constipation management.

Participants receiving *Lactiplantibacillus plantarum* Probio87 demonstrated significant and sustained improvements across all domains of the PAC-QOL, reflecting not only relief of physical symptoms but also meaningful enhancement in emotional and psychosocial well-being. These findings are consistent with prior studies reporting that probiotic supplementation can alleviate constipation-related discomfort and improve perceived quality of life through modulation of gut function and mood regulation (12).

The observed reduction in PAC-QOL total and subscale scores suggests that *L. plantarum* Probio87 alleviated abdominal discomfort and concerns related to bowel habits, thereby improving daily functioning and overall satisfaction. Beyond local intestinal effects, accumulating evidence indicates that probiotics may influence host psychological states via the gut–brain axis. In particular, reductions in intestinal inflammation and improved serotonergic signaling can contribute to diminished anxiety and mood disturbances associated with chronic constipation (5, 31, 32, 44).

The persistence of improved PAC-QOL scores during the post-intervention follow-up highlights the stability of these benefits, suggesting that microbial and neuromodulatory adaptations induced by *L. plantarum* Probio87 may continue beyond the supplementation period. Clinically, this sustained improvement underscores the potential of targeted probiotic therapy as a safe and practical adjunct to enhance patient-reported outcomes, complementing standard lifestyle and pharmacologic measures for functional constipation management.

This trial demonstrated that daily supplementation with *Lactiplantibacillus plantarum* Probio87 was associated with significant improvement in psychological well-being, as reflected by reduced HAMD-24 scores, particularly in anxiety, somatization and sleep-related domains. These results complement previous clinical studies reporting mood-enhancing and stress-modulating effects of probiotics in patients with gastrointestinal disorders (45). The observed psychological improvement may be mediated through gut–brain axis modulation, whereby restoration of intestinal homeostasis and reduction of low-grade inflammation contribute to enhanced mood and sleep quality. In this study, the down-regulation of pro-inflammatory genes such as IL-1β and IDO further supports the hypothesis that alleviation of inflammatory signaling may underpin the improved emotional state, consistent with the inflammation-related model of depression (46). Moreover, the parallel improvement in PAC-QOL domains suggests that relief of constipation-related physical discomfort and improved stool regularity may secondarily reduce psychological burden and sleep disruption. Overall, the psychological benefits observed in this trial align with the growing evidence that specific *L. plantarum* strains can positively influence mood regulation via neuroimmune and metabolic pathways along the gut–brain axis (47). While the current findings are encouraging, larger multicenter trials incorporating validated psychometric and neuroimaging measures are warranted to confirm the causal relationship between gut microbial modulation and mental health improvement in functional constipation.

From a nutritional perspective, Probio87 supplementation may be viewed as a microbiota-targeted dietary strategy that complements standard constipation management, including dietary fiber intake, hydration, and physical activity. The clinical improvements observed under standardized lifestyle recommendations suggest that the probiotic effect was not simply attributable to general lifestyle advice, because both groups received the same recommendations. Instead, the findings support the potential additive value of targeted probiotic supplementation within a structured lifestyle-management framework. This is particularly relevant for functional constipation, where long-term pharmacological treatment may be undesirable and patient-centered nutritional strategies are often preferred.

The exploratory whole-blood gene-expression findings provide supportive evidence that clinical improvement after *Lactiplantibacillus plantarum* Probio87 supplementation was accompanied by changes in peripheral host-response pathways. Because gene expression was measured in whole blood rather than intestinal mucosal tissue, these results should be interpreted cautiously as systemic transcriptional signals rather than direct evidence of local gut inflammation, mucosal immunity, or enteric neuromuscular regulation.

In the present analysis, BDNF and SSTR expression were both higher in the probiotic group than in the placebo group at baseline (*p* = 0.040 and *p* = 0.049, respectively), while MLNR expression was lower in the probiotic group at baseline (*p* = 0.007). Therefore, BDNF, SSTR, and MLNR should not be interpreted as intervention-induced transcriptional responses. Instead, the attenuation of these baseline differences may reflect normalization of between-group variability over the course of supplementation. The baseline pattern of lower MLNR and higher SSTR expression in the probiotic group is biologically coherent with a more pronounced constipation phenotype at enrollment. MLNR activation enhances propulsive contractions and accelerates gut transit, whereas SSTR activation inhibits peristalsis and prolongs stool transit (48, 49). The convergence of these between-group differences at Week 8 may suggest that probiotic supplementation was associated with a degree of normalisation in gut motility signalling, contributing to the observed improvement in bowel movement frequency.

After 8 weeks of intervention, the probiotic group showed lower expression of IL-1β and IDO1 compared with the placebo group. The reduced expression of IL-1β suggests attenuation of peripheral pro-inflammatory signaling, which may be consistent with a less inflammatory host-response profile accompanying symptom improvement. IDO1 is involved in the tryptophan–kynurenine pathway and is often induced by inflammatory signaling. Therefore, lower IDO1 expression may suggest reduced activation of inflammation-related tryptophan metabolism, although the present study did not directly measure tryptophan, kynurenine, serotonin, or related metabolites.

The probiotic group also showed lower expression of CD117 and CXCR5 after intervention. These genes are related to immune-cell activation, migration, and trafficking. Their reduced expression should not be interpreted as broad immune suppression, but rather as a possible shift toward a less activated peripheral immune profile. This interpretation is consistent with the observed reduction in inflammatory signaling, but further immune phenotyping would be required to clarify the cellular basis of these changes.

In addition, lower expression of DBH was observed in the probiotic group after 8 weeks. This findings indicate that neuroendocrine-associated transcriptional signals were altered following probiotic supplementation. However, because DBH and MLNR expression was measured in whole blood, these changes cannot be taken as direct evidence of altered catecholamine metabolism or intestinal motility receptor activity. Confirmation using circulating metabolites, gut hormones, protein-level assays, or intestinal tissue-based analyses would be required.

Overall, these gene-expression findings suggest that Probio87 supplementation was associated with coordinated peripheral transcriptional changes involving inflammatory, immune, neuroendocrine, and motility-related pathways. These changes provide a biologically plausible host-response context for the observed improvements in bowel function, quality of life, and psychological well-being. Nevertheless, they remain exploratory and should not be interpreted as definitive mechanistic evidence.

In this randomized, double-blind, placebo-controlled trial, *Lactiplantibacillus plantarum* Probio87 demonstrated excellent genomic and clinical safety. Whole-genome analysis confirmed the absence of virulence or antimicrobial-resistance genes, which meets the Qualified Presumption of Safety (QPS) standards established by the European Food Safety Authority (EFSA, 2020). These results are consistent with previous genomic evaluations of *L. plantarum* strains, which have shown a lack of transferable antibiotic-resistance determinants and an overall safe genetic background (50).

Clinical laboratory results further supported its safety, as no significant deviations in hematological or biochemical parameters were observed throughout the intervention period. These findings are in line with earlier human studies, including Nisaa et al. (19), where Probio87 supplementation was well tolerated and associated with no adverse clinical outcomes. Similar conclusions were also reported in other probiotic trials, where *L. plantarum* species were shown to be safe and well tolerated during long-term use (51).

Collectively, these results confirm that *L. plantarum* Probio87 meets internationally accepted safety criteria and can be considered a safe probiotic candidate for human application.

This study has several strengths, including its randomized, double-blind, placebo-controlled design, standardized lifestyle recommendations across both groups, longitudinal clinical follow-up, and integrated assessment of clinical outcomes, peripheral gene-expression profiles, and strain safety. These features provide a comprehensive evaluation of *Lactiplantibacillus plantarum* Probio87 as a probiotic supplementation strategy for functional constipation.

Several limitations should also be acknowledged. First, gene expression was assessed in whole blood rather than intestinal mucosal tissue; therefore, the findings represent peripheral host-response signals rather than direct evidence of local intestinal mechanisms. Second, gene expression was measured only at W0 and W8, which precluded assessment of whether these transcriptional changes persisted during the post-intervention follow-up. Third, the targeted qPCR panel was exploratory and was not supported by protein-level validation or direct quantification of related metabolites, neurotransmitter precursors, or gut hormones. Fourth, although lifestyle recommendations were standardized across groups, detailed quantitative dietary intake was not recorded. Finally, this was a single-center trial, and larger multicenter studies are needed to confirm the clinical and molecular findings.

## 5 Conclusion

In this randomized, double-blind, placebo-controlled trial, *Lactiplantibacillus plantarum* Probio87 significantly improved bowel function in adults with functional constipation, as evidenced by increased complete spontaneous bowel movements and reduced constipation symptom severity. Supplementation was also associated with improvements in constipation-related quality of life and psychological well-being, with benefits persisting during the post-intervention follow-up period. Exploratory whole-blood gene-expression analyses identified changes in inflammatory, immune, neuroendocrine, and motility-related pathways, suggesting potential host-response mechanisms associated with the clinical improvements. Furthermore, Probio87 demonstrated a favorable safety profile, with no intervention-related adverse events or clinically relevant abnormalities observed. These findings support the potential of *L. plantarum* Probio87 as a safe adjunctive nutritional strategy for the management of functional constipation.

## Data Availability

The datasets presented in this study can be found in online repositories. The names of the repository/repositories and accession number(s) can be found in the article/Supplementary material.
