# Supplementary material for: Lactiplantibacillus plantarum Probio87 supplementation improves functional constipation and is associated with peripheral gene-expression responses related to inflammation and the gut–brain axis: a randomized, double-blind, placebo-controlled trial
Source: Front Nutr. 2026 Jul 17;13:1876944. doi: 10.3389/fnut.2026.1876944 (PMC13426275; doi:10.3389/fnut.2026.1876944)
Supplement: Supplementary file 1 [file Table_1.docx]

**Supplementary Table S1. Primer sequences and references for target genes used in qPCR analysis.**

| Target gene | Primer sequence (5′–3′) | Reference |
| --- | --- | --- |
| BDNF | F: TAACGGCGGCAGACAAAAAGA R: GAAGTATTGCTTCAGTTGGCCT | [36] |
| IL-1β | F: GACCACCACTACAGCAAGG  R: AAAGATGAAGGGAAAGAAGGT | NM_000787 (Homo sapiens dopamine beta-hydroxylase [IL-1β] mRNA, NCBI RefSeq record). |
| CD117 | F: CCAGAGTGCTCTAATGACTG R: AGCCCCTGTTTCATACTGAC | [37] |
| CXCR5 | F: GCTAACGCTGGAAATGGA R: GCAGGGCAGAGATGATTT | [38] |
| DBH | F: GGTGATAGAAGGACGAAACGA R: AGTAGCCAGTGAGGATGAAGG | NM_000787 (Homo sapiens dopamine beta-hydroxylase [DBH] mRNA, NCBI RefSeq record). |
| IDO | F: TTCCTGGTCTCTCTATTGGTG R: TAGCAAAGTGTCCCGTTCTT | NM_002164 (Homo sapiens indoleamine 2,3-dioxygenase 1 [IDO1] mRNA, NCBI RefSeq record). |
| MLNR | F: CGCATCTATCAACCCAATCC R: ACCGTGTCTCCTCCAGTGTC | NM_001507.1 (Homo sapiens motilin receptor [MLNR] mRNA, NCBI RefSeq record). |
| SSTR | F: CCTGGAGACCACAAATCAA R: AATGGCGAAAAGAAACACA | NM_001050.3 (Homo sapiens somatostatin receptor 2 [SSTR2] mRNA, NCBI RefSeq record). |
| 18S rRNA | F: CTCAACACGGGAAACCTCAC R: CGCTCCACCAACTAAGAACG | [39] |
| GAPDH | F: TGGGTGTGAACCACGAGAA R: GGCATGGACTGTGGTCATGA | [40] |
| β-actin | F: CCTGGCACCCAGCACAAT R: GGGCCGGACTCGTCATAC | [41] |

BDNF: Brain-Derived Neurotrophic Factor; CD117: KIT Proto-Oncogene, Receptor Tyrosine Kinase; CXCR5: C-X-C Motif Chemokine Receptor 5; DBH: Dopamine Beta-Hydroxylase;
IDO: Indoleamine 2,3-Dioxygenase; MLNR: Motilin Receptor; SSTR2: Somatostatin Receptor. 18S rRNA: 18S ribosomal RNA; β-Actin: beta-actin; GAPDH: Glyceraldehyde-3-Phosphate Dehydrogenase.

**Supplementary Table S2.** PAC-QOL items (mean ± SE) for patients with functional constipation and administered with placebo (n=50) or probiotic (n=51) for 8-weeks (W0-W8) followed by a 4-week post-intervention observational period (W8-W12).

| **Domain** | **Placebo** | | | | | **Probiotic** | | | | | | **Between-group** | | |
| --- | --- | --- | --- | --- | --- | --- | --- | --- | --- | --- | --- | --- | --- | --- |
|  | **W0** | **W8** | **W12** | **P-value** | | **W0** | **W8** | **W12** | **P-value** | | **P-value** | | | |
|  |  |  |  | **W0–W8** | **W8–W12** |  |  |  | **W0–W8** | **W8–W12** | **W0** | | **W8** | **W12** |
| Physical Discomfort | 5.42±0.46 | 4.94±0.41 | 4.74±0.41 | 0.006 | 0.222 | 4.94±0.44 | 1.73±0.23 | 0.96±0.15 | 0.006 | 0.000 | 0.399 | | 0.000 | 0.000 |
| Psychological Discomfort | 10.30 ± 0.95 | 8.90 ± 0.92 | 8.64 ± 0.88 | 0.000 | 0.166 | 7.24 ± 0.76 | 2.98 ± 0.49 | 2.06 ± 0.42 | 0.000 | 0.000 | 0.022 | | 0.000 | 0.000 |
| Worries and concerns | 20.96 ± 1.53 | 18.54 ± 1.46 | 17.44 ± 1.42 | 0.000 | 0.440 | 20.61 ± 1.60 | 7.33 ± 0.86 | 4.41 ± 0.75 | 0.000 | 0.000 | 0.796 | | 0.000 | 0.000 |
| Dissatisfaction | 18.04 ± 0.50 | 15.14 ± 0.65 | 14.84 ± 0.72 | 0.000 | 0.505 | 17.71 ± 1.06 | 6.86 ± 0.66 | 4.67 ± 0.55 | 0.000 | 0.000 | 0.223 | | 0.000 | 0.000 |
| Total | 54.72 ± 2.77 | 47.52 ± 2.83 | 45.66 ± 2.80 | 0.000 | 0.100 | 50.49 ± 3.16 | 18.90 ± 1.87 | 12.16 ± 1.58 | 0.000 | 0.000 | 0.210 | | 0.000 | 0.000 |

**Supplementary Table S3.** Hamilton Depression Rating Scale- 24 (HAMD-24) domain scores (mean ± SE) for patients with functional constipation and administered with placebo (n=50) or probiotic (n=51) for 8-weeks (W0-W8) followed by a 4-week post-intervention observational period (W8-W12).

| **Domain** | **Placebo** | | | | | | | | **Probiotic** | | | | | **Between-group** | | |
| --- | --- | --- | --- | --- | --- | --- | --- | --- | --- | --- | --- | --- | --- | --- | --- | --- |
|  | **W0** | | **W8** | **W12** | | **P-value** | | | **W0** | **W8** | **W12** | **P-value** | | **P-value** | | |
|  |  |  |  |  |  | **W0–W8** | | **W8–W12** |  |  |  | **W0–W8** | **W8–W12** | **W0** | **W8** | **W12** |
| Anxiety | 1.60 ± 0.28 | 1.38 ± 0.28 | | | 1.40 ± 0.24 | | 0.064 | 0.868 | 1.78 ± 0.23 | 0.49 ± 0.14 | 0.71 ± 0.20 | <0.001 | 0.415 | 0.434 | <0.001 | 0.001 |
| Sleep | 2.06 ± 0.20 | 1.98 ± 0.18 | | | 1.94 ± 0.17 | | 0.472 | 0.868 | 1.88 ± 0.20 | 1.16 ± 0.17 | 1.29 ± 0.18 | <0.001 | 0.127 | 0.584 | 0.001 | 0.006 |
| Weight | 0.04 ± 0.03 | 0.06 ± 0.03 | | | 0.02 ± 0.02 | | 0.564 | 0.157 | 0.06 ± 0.03 | 0.00 ± 0.00 | 0.02 ± 0.02 | 0.083 | 0.317 | 0.681 | 0.077 | 0.989 |
| Cognitive disturbance | 0.50 ± 0.13 | 0.38 ± 0.12 | | | 0.30 ± 0.12 | | 0.034 | 0.046 | 0.49 ± 0.21 | 0.16 ± 0.08 | 0.10 ± 0.06 | 0.019 | 0.257 | 0.336 | 0.093 | 0.101 |
| Diurnal variation | 0.28 ± 0.08 | 0.20 ± 0.07 | | | 0.18 ± 0.06 | | 0.102 | 0.564 | 0.20 ± 0.08 | 0.10 ± 0.04 | 0.12 ± 0.05 | 0.059 | 0.317 | 0.220 | 0.326 | 0.518 |
| Retardation | 0.80 ± 0.17 | 0.66 ± 0.18 | | | 0.74 ± 0.17 | | 0.051 | 0.234 | 0.69 ± 0.14 | 0.24 ± 0.10 | 0.27 ± 0.10 | <0.001 | 0.414 | 0.856 | 0.041 | 0.011 |
| Hopelessness | 0.50 ± 0.15 | 0.38 ± 0.15 | | | 0.32 ± 0.13 | | 0.271 | 0.083 | 0.47 ± 0.16 | 0.14 ± 0.06 | 0.10 ± 0.04 | 0.011 | 0.414 | 0.785 | 0.428 | 0.589 |
| Total | 6.40 ± 0.78 | 5.36 ± 0.83 | | | 5.36 ± 0.70 | | 0.001 | 0.977 | 5.98 ± 0.79 | 2.37 ± 0.45 | 2.61 ± 0.43 | <0.001 | 0.038 | 0.524 | <0.001 | <0.001 |

**Supplementary Table S4.** Antimicrobial Resistance and Virulence Gene Annotation Results.

| **Category** | **Database** | **Target/GeneClass** | **Result** | **%Identity** | **%Coverage** | **Interpretation**  **(Polished,EN)** |
| --- | --- | --- | --- | --- | --- | --- |
| Antibiotic Resistance | CARD | D-Ala-D-Ala carboxypeptidase VanY (vanY; VanB-type operon) | Hit | 31.93 | 95.9 | Low-identity CARD hits likely reflect background homology, not a functional acquired resistance gene. |
|  | CARD | Quaternary Ammonium Compound resistance efflux pump QacJ (qacJ; SMR family) | Hit | 40.20 | 95.33 | Low-identity CARD hits likely reflect background homology, not a functional acquired resistance gene. |
|  | ResFinder | All acquired antimicrobial resistance genes | No Hits | N/A | N/A | No acquired antimicrobial resistance genes were identified. |
| Virulence | VFDB | Elongation factor Tu (EF-Tu; tufA) | Hit | 71.00 | 100 | These VFDB hits are conserved; single-gene matches don’t imply pathogenicity. |
|  | VFDB | UTP-glucose-1-phosphate uridylyltransferase (GalU/HasC; hasC) | Hit | 75.30 | 100 | These VFDB hits are conserved; single-gene matches don’t imply pathogenicity. |
|  | VFDB | ATP-dependent Clp protease proteolytic subunit (ClpP; clpP) | Hit | 70.40 | 98.4 | These VFDB hits are conserved; single-gene matches don’t imply pathogenicity. |
|  | VirulenceFinder | All virulence gene markers | No Hits | N/A | N/A | No virulence genes were identified |

**Supplementary Table S5.** PathogenFinder2 result for Probio87.

| **Genome** | **Prediction_0** | **Prediction_1** | **Prediction_2** | **Prediction_3** | **PredictionMean** | **PredictionSTD** | **Phenotype** |
| --- | --- | --- | --- | --- | --- | --- | --- |
| Probio87 | 0.017379761 | 0.028869629 | 0.176391602 | 0.029373169 | 0.06300354 | 0.065640132 | Human Non Pathogenic |

Values represent the predicted probability of a strain being pathogenic (range: 0-1), as determined by PathogenFinder bioinformatic analysis.

**Supplementary Table S6.** Blood clinical pathology safety indicators (mean ± SE) for patients with functional constipation and administered with placebo (n=50) or probiotic (n=51) for 8-weeks.

| **Indicator** | **Placebo W0** | **Probiotic W0** | **P-value** | **Placebo W8** | **Probiotic W8** | **P-value** |
| --- | --- | --- | --- | --- | --- | --- |
| Full Blood Count |  |  |  |  |  |  |
| White Blood Cells (10^9 cells/L) | 5.77 ± 0.23 | 5.44 ± 0.21 | 0.421 | 5.65 ± 0.20 | 5.39 ± 0.22 | 0.263 |
| Lymphocyte (%) | 33.05 ± 1.59 | 32.39 ± 1.36 | 0.666 | 32.95 ± 1.46 | 33.47 ± 1.69 | 0.406 |
| Lymphocyte (10^9 cells/L) | 2.28 ± 0.44 | 1.73 ± 0.08 | 0.201 | 1.81 ± 0.08 | 1.84 ± 0.07 | 0.677 |
| Monocyte (%) | 6.48 ± 0.24 | 6.32 ± 0.24 | 0.960 | 6.61 ± 0.24 | 6.26 ± 0.36 | 0.405 |
| Monocyte (10^9 cells/L) | 0.47 ± 0.10 | 0.50 ± 0.15 | 0.513 | 0.37 ± 0.02 | 0.35 ± 0.02 | 0.224 |
| Neutrophil (%) | 58.12 ± 1.17 | 58.66 ± 1.5 | 0.692 | 57.60 ± 1.66 | 54.77 ± 1.87 | 0.337 |
| Neutrophil (10^9 cells/L) | 3.43 ± 0.22 | 3.32 ± 0.18 | 0.892 | 3.31 ± 0.17 | 3.09 ± 0.17 | 0.316 |
| Eosinophil (%) | 2.16 ± 0.43 | 1.97 ± 0.3 | 0.865 | 2.27 ± 0.39 | 1.95 ± 0.25 | 0.788 |
| Eosinophil (10^9 cells/L) | 0.13 ± 0.03 | 0.10 ± 0.01 | 0.943 | 0.17 ± 0.03 | 0.11 ± 0.01 | 0.788 |
| Basophil (%) | 0.54 ± 0.04 | 0.52 ± 0.04 | 0.789 | 0.58 ± 0.04 | 0.57 ± 0.05 | 0.570 |
| Basophil (10^9 cells/L) | 0.11 ± 0.09 | 0.05 ± 0.02 | 0.394 | 0.03 ± 0.00 | 0.03 ± 0.00 | 0.518 |
| Red Blood Cells (10^12 cells/L) | 4.37 ± 0.05 | 4.51 ± 0.05 | 0.107 | 4.35 ± 0.05 | 4.49 ± 0.05 | 0.104 |
| Hemoglobin (g/L) | 130.15 ± 1.69 | 132.45 ± 1.95 | 0.356 | 128.28 ± 1.69 | 131.19 ± 1.71 | 0.183 |
| Hematocrit (%) | 40.72 ± 0.47 | 41.68 ± 0.47 | 0.068 | 40.39 ± 0.50 | 41.17 ± 0.48 | 0.258 |
| Mean Corpuscular Volume (fL) | 93.30 ± 0.76 | 92.58 ± 0.83 | 0.979 | 93.00 ± 0.68 | 91.97 ± 0.90 | 0.788 |
| Mean Corpuscular Hemoglobin (pg) | 29.84 ± 0.32 | 29.41 ± 0.33 | 0.518 | 29.56 ± 0.30 | 29.31 ± 0.34 | 0.961 |
| Mean Corpuscular Hemoglobin Concentration (g/L) | 319.49 ± 1.79 | 317.43 ± 1.46 | 0.200 | 317.71 ± 1.45 | 318.48 ± 1.23 | 0.889 |
| Red Cell Distribution Width - Standard Deviation (fL) | 45.21 ± 0.56 | 44.21 ± 0.54 | 0.178 | 44.90 ± 0.50 | 44.53 ± 0.45 | 0.997 |
| Red Cell Distribution Width - Coefficient of Variation (%) | 13.25 ± 0.19 | 13.15 ± 0.18 | 0.449 | 13.16 ± 0.16 | 13.29 ± 0.2 | 0.604 |
| Platelet Count (10^9 platelets/L) | 219.00 ± 9.34 | 220.49 ± 8.93 | 0.675 | 219.00 ± 10.18 | 224.07 ± 8.87 | 0.922 |
| Liver Function Tests |  |  |  |  |  |  |
| Total Bilirubin (μmol/L) | 12.72 ± 0.65 | 13.23 ± 0.60 | 0.477 | 12.40 ± 0.49 | 13.84 ± 0.86 | 0.284 |
| Direct Bilirubin (μmol/L) | 4.10 ± 0.21 | 4.28 ± 0.21 | 0.310 | 4.28 ± 0.18 | 4.55 ± 0.31 | 0.922 |
| Total Protein (g/L) | 71.86 ± 0.80 | 72.92 ± 0.63 | 0.151 | 72.30 ± 0.69 | 72.96 ± 0.50 | 0.081 |
| Albumin (g/L) | 45.41 ± 0.37 | 46.10 ± 0.36 | 0.294 | 45.43 ± 0.32 | 45.79 ± 0.25 | 0.360 |
| Globulin (g/L) | 26.31 ± 0.61 | 26.71 ± 0.49 | 0.324 | 26.95 ± 0.60 | 27.03 ± 0.42 | 0.388 |
| Albumin/Globulin Ratio | 1.77 ± 0.04 | 1.75 ± 0.03 | 0.530 | 1.73 ± 0.04 | 1.71 ± 0.03 | 0.573 |
| Total Bile Acids (μmol/L) | 11.82 ± 7.70 | 11.12 ± 7.36 | 0.829 | 3.76 ± 0.58 | 4.46 ± 0.82 | 0.127 |
| Alanine Aminotransferase (U/L) | 17.28 ± 1.40 | 21.39 ± 2.23 | 0.278 | 19.24 ± 2.00 | 17.71 ± 1.22 | 0.699 |
| Aspartate Aminotransferase (U/L) | 20.68 ± 0.78 | 22.30 ± 0.88 | 0.343 | 21.09 ± 0.83 | 21.23 ± 0.73 | 0.389 |
| Gamma-Glutamyl Transferase (U/L) | 19.37 ± 1.95 | 17.19 ± 1.24 | 0.957 | 18.93 ± 2.16 | 18.31 ± 1.73 | 0.264 |
| Alkaline Phosphatase (U/L) | 61.00 ± 3.20 | 61.69 ± 2.79 | 0.727 | 66.15 ± 2.85 | 62.67 ± 2.93 | 0.342 |
| Lactate Dehydrogenase (U/L) | 171.41 ± 8.66 | 186.61 ± 9.06 | 0.346 | 188.55 ± 8.03 | 188.77 ± 6.16 | 0.235 |
| Glucose (mmol/L) | 5.16 ± 0.22 | 5.13 ± 0.20 | 0.482 | 4.94 ± 0.19 | 5.05 ± 0.16 | 0.385 |
| Kidney Function Tests |  |  |  |  |  |  |
| Urea (mmol/L) | 6.76 ± 1.46 | 5.48 ± 0.20 | 0.905 | 6.34 ± 1.09 | 7.59 ± 2.07 | 0.484 |
| Creatinine (μmol/L) | 65.18 ± 4.81 | 60.33 ± 1.76 | 0.477 | 65.42 ± 5.46 | 68.17 ± 7.81 | 0.899 |
| Uric acid (μmol/L) | 286.24 ± 11.90 | 333.27 ± 49.86 | 0.925 | 290.20 ± 7.14 | 284.18 ± 11.59 | 0.836 |
| Estimated Glomerular Filtration Rate (mL/min) | 110.78 ± 6.74 | 103.30 ± 2.38 | 0.900 | 107.65 ± 1.83 | 102.36 ± 3.92 | 0.898 |
